# Spontaneous Regression of Cardiac Rhabdomyoma Presenting as Severe Left Ventricular Inlet Obstruction in a Neonate with Tuberous Sclerosis

**DOI:** 10.1155/2018/8395260

**Published:** 2018-01-28

**Authors:** Eun Song Song, Kumi Jeong, Gun Kim, In Ji Hwang, Mi-Ji Lee, Hwa Jin Cho, Young Kuk Cho

**Affiliations:** Department of Pediatrics, Chonnam National University Medical School, Chonnam National University Hospital, Gwangju, Republic of Korea

## Abstract

Cardiac rhabdomyoma can be subclinical or have a fatal presentation according to the onset age and involved site, size, and degree of invasion. Although most cardiac rhabdomyomas become smaller with time, emergency intervention is indicated when severe obstruction has occurred. In this report, we describe the spontaneous regression of a large cardiac rhabdomyoma (20.5 × 15.6 mm) presenting as severe left ventricular inlet obstruction in a neonate with tuberous sclerosis. Although a cardiac rhabdomyoma can be large enough to induce left ventricular inlet obstruction, conservative treatment without aggressive surgical intervention can be considered if the hemodynamic condition does not deteriorate.

## 1. Introduction

Rhabdomyoma is the most common cardiac tumor in infants and children and is reported in up to 50–64% of infants with tuberous sclerosis [1, 2]. Its expression ranges from subclinical to fatal presentation, according to the onset age and the site, size, and degree of invasion [3]. Although rhabdomyomas become smaller with time, when severe obstruction induces hemodynamic instability or a rhythm abnormality, emergency intervention is indicated [4–6]. Rhabdomyoma causing left ventricular (LV) inlet obstruction can necessitate an emergency operation [3, 7–10]. However, there is no recorded report of spontaneous resolution of a huge cardiac rhabdomyoma causing LV inlet obstruction. We report a case of spontaneous regression of a large symptomatic cardiac rhabdomyoma presenting as severe LV inlet obstruction, occupying 75–90% of the mitral valve (MV) annulus in a neonate with tuberous sclerosis.

## 2. Case Presentation

A male infant was born at 39 weeks of gestation by vaginal delivery to a healthy 35-year-old female. There was no family history of congenital heart disease or tuberous sclerosis. At 31 weeks of gestation, a fetal echocardiogram revealed a large, solitary LV mass and multiple right ventricular (RV) masses. The delivery was uneventful. The Apgar score was 10 at both 1 and 5 minutes. Birth weight of 3,450 g, length of 51.0 cm, and head circumference of 35.5 cm were normal. Transcutaneous oxygen saturation was 95% on room air, heart rate was 132 bpm, respiratory rate was 48/minute, arterial pressure was 62/38 mmHg, a grade 2/6 mid-diastolic rumbling murmur was heard at the left sternal border, and there were no skin lesions. Chest X-ray showed mild cardiomegaly with increased pulmonary vascular markings. Transcranial ultrasonography was normal.

**Figure 1:**
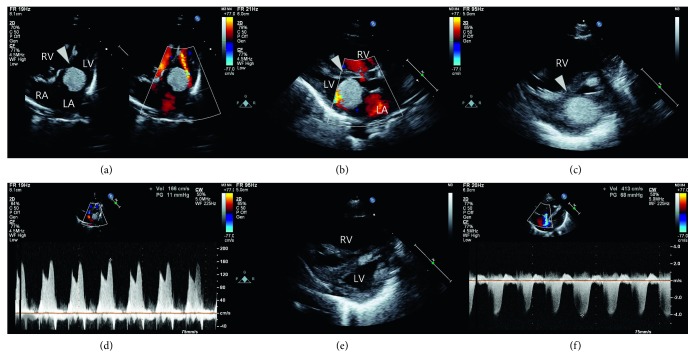
Two-dimensional echocardiography shows a large rhabdomyoma (arrowhead), 20.5 × 15.6 mm in diameter, occupying 75–90% of the mitral annulus, in addition to multiple small tubers in both ventricles and the left ventricular outflow tract after birth in apical 4-chamber (a), parasternal long-axis (b), and parasternal short-axis (c) views. Right atrial, right ventricular (RV), and left atrial dilatation were also observed. Pulse Doppler echocardiography showed a peak velocity through the mitral valve of 1.66 m/s (d). RV dilatation and pressure overload secondary to pulmonary hypertension were observed in a parasternal, end-diastolic short-axis view (e). The peak velocity through the tricuspid valve was 4.13 m/s (f).

Two-dimensional echocardiographic examination revealed a large solitary tumor measuring 20.5 × 15.6 mm and occupying 75–90% of the mitral annulus (Figures 1). This tumor was attached to the anterior leaflet of the MV, causing LV inlet obstruction. The peak velocity through the MV was 1.66 m/s (Figure 1). Right atrial and RV dilatation and pressure overload secondary to pulmonary hypertension were observed (Figure 1). The peak jet velocity through the regurgitant tricuspid valve was 4.13 m/s (Figure 1). Mild mitral regurgitation (MR), left atrial (LA) enlargement without LV dilatation, and multiple small tubers in both ventricles and the LV outflow tract were observed (Figures 1). LV ejection fraction was normal at 67.2%. Although the newborn had mild subcostal chest retractions, his vital signs were stable; therefore, inotropes and oxygen were not provided. At 5 h of age, paroxysmal supraventricular tachyarrhythmia at 240 bpm developed. Because intermittent supraventricular tachyarrhythmia with desaturation and irritability continued, repeated doses of adenosine were administered intravenously to restore sinus rhythm. Digoxin was started for long-term management and the tachyarrhythmia resolved. Genetic analysis was performed to detect mutations of tuberous sclerosis complex (*TSC*) 1 and 2 genes. *TSC1* mutation was absent, but a *TSC2* mutation (*c.5161*–*1G* *>* *C*) was detected. Ophthalmic examination revealed a jagged-edged, linear depigmented retina in the right eye. During the 14-day hospital stay, blood pressure was well maintained without inotropes, tachypnea improved, the frequency of breast-feeding increased, and weight gain was observed. Follow-up chest X-ray showed regression of cardiomegaly and pulmonary vascular markings. Follow-up echocardiography demonstrated decreased MR and pulmonary hypertension and normal systolic LV function. The infant was discharged with only digoxin to prevent arrhythmias.

**Figure 2:**
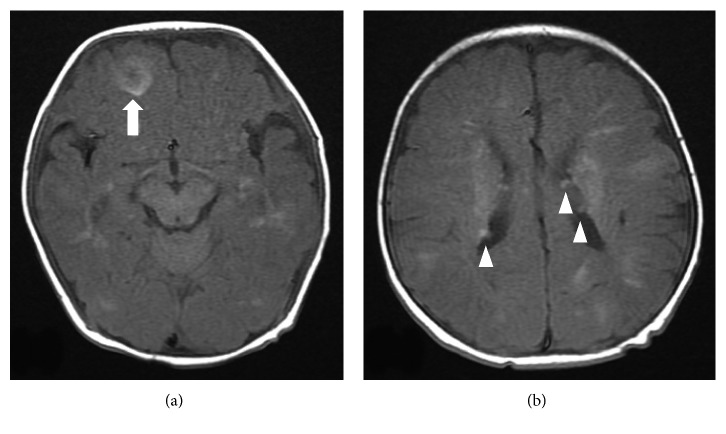
At 3 months of age, T1-weighted magnetic resonance imaging showed a right frontal cortical tuber (arrow) (a) and high signal intensity lesions in both cerebral hemispheres, with multiple nodular lesions (arrowheads) along the lateral ventricular caudothalamic grooves (b).

At 40 days of age, symptoms and signs associated with LV inlet obstruction and tachyarrhythmia were absent. Echocardiography showed good LV function with decreased rhabdomyoma size (16.8 × 11.5 mm). At 3 months of age, a tonic-type seizure occurred and right frontal cortical tubers, multifocal white-matter lesions, and subependymal nodules were discovered on brain magnetic resonance imaging (Figures 2(a) and 2(b)). Vigabatrin as an antiepileptic drug was started. Depigmented skin lesions were also found that time on the right shoulder and chest.

**Figure 3:**
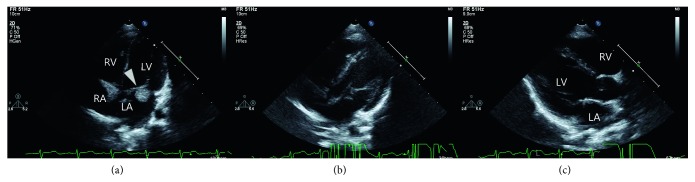
Two-dimensional follow-up echocardiography showed that rhabdomyoma size was significantly decreased at 6 months of age (a). At 3 years of age, a very small (6.5 × 2.3 mm), ellipse-shaped rhabdomyoma attached to the MV anterior leaflet without LV inlet obstruction (peak velocity = 0.76 m/sec) (b and c) was observed.

At 6 months of age, echocardiography revealed a further decrease in rhabdomyoma size (13.8 × 12.1 mm); the lesion occupied a smaller portion (about 50%) of the mitral annulus (Figure 3). Tricuspid regurgitation and pulmonary hypertension were improved. MR and LA dilatation were not observed. Digoxin was maintained up to 24 months of age. After discontinuation of medication, no further tachyarrhythmias occurred.

At 3 years of age, echocardiography showed a very small (6.5 × 2.3 mm), ellipse-shaped rhabdomyoma attached to the MV anterior leaflet without LV inlet obstruction (peak velocity = 0.76 m/s) (Figures 3 and 3). At that time, fibroadenomas began to develop on the face. Convulsions were controlled by vigabatrin and topiramate. A developmental delay of about 18 months was apparent.

## 3. Discussion

Although cardiac rhabdomyomas are known to spontaneously regress in patients with tuberous sclerosis, many complications associated with tumor location and size are possible [1–3]. Some patients are very symptomatic, with signs of severe heart failure or valve dysfunction due to blood flow obstruction, and even univentricular physiology [7]. Only five case reports of hemodynamically significant LV inflow obstruction caused by cardiac rhabdomyoma have been published (Table 1). Four cases were associated with tuberous sclerosis [3, 7, 9, 11], and most were detected in infancy and the perinatal period. The other case involved a tumor detected in adulthood. Common initial symptoms were related to heart failure, including cyanosis and dyspnea with or without cardiomegaly [9]. Surgical resection of tumor was performed in four patients in whom the lesions were attached to the septum. A 1-day-old infant underwent atrial septostomy by cardiac catheterization due to hemodynamic instability.

In our case, a large rhabdomyoma (20.5 × 15.6 mm) occupied 75–90% of the LV inlet and caused obstruction, LA enlargement, and pulmonary hypertension. The patient did not undergo surgical intervention because he was hemodynamically stable. Since the rhabdomyoma was attached to the anterior leaflet of the MV, there was a risk of valve dysfunction after surgery. With increasing age, the LV inflow obstruction was attenuated by decreasing rhabdomyoma and increasing mitral annulus size. At 3 years of age, the rhabdomyoma had decreased to an ellipse-shaped mass 6.5 × 2.3 mm in size and showed no LV flow disturbance. Repeat echocardiography to confirm hemodynamic stability played a critical role in determining treatment. Many reports have emphasized the usefulness of echocardiography in the management of cardiac rhabdomyoma in neonates or children. Di Liang et al. [11] described serial echocardiographic evaluation of cardiac rhabdomyoma. Follow-up echocardiography revealed that of 22 tumors in 8 patients, 7 completely regressed, 7 partially regressed, and 8 remained stable. In addition, Batmaz et al. [12] reported a huge spherical rhabdomyoma originating from the MV in an asymptomatic patient with a murmur and experienced spontaneous regression with conservative treatment alone. In the present case, the neonate had an arrhythmia and evidence of congestive heart failure. Hemodynamic stability was confirmed accurately and continuously with echocardiography. Therefore, we provided conservative treatment.

## 4. Conclusion

Although a cardiac rhabdomyoma can be large enough to induce LV inlet obstruction, conservative treatment without aggressive surgical intervention can be considered if hemodynamic conditions do not deteriorate. In addition, repeated echocardiography may play a pivotal role in understanding hemodynamic status and establishing a treatment plan.

## Figures and Tables

**Table 1:** Clinical features of patients with significant LV inlet obstruction caused by cardiac rhabdomyoma.

Reference, year	Age at diagnosis	Initial presentation	Rhabdomyoma number	Rhabdomyoma treatment	Spontaneous rhabdomyoma regression	Genetic analysis	Tuberous sclerosis-associated sign	Outcome
Mair et al., 1977 [7]	1 day	Cyanosis	Multiple	Atrial septostomy	No	None	Cerebral hamartoma	Died
		Tachypnea						
		Tachycardia						
		Cardiomegaly						
		Hepatomegaly						
Muhler et al., 1994 [3]	5 weeks	Pulmonary edema	1	Tumor resection	No	None	None	Died
Dyamenahalli et al., 1998 [8]	1 day	Cyanosis	1	Tumor resection	No	None	Unknown	Alive
		Tachycardia						
Abdel-Rahman et al., 2005 [9]	Prenatal	Cyanosis	3	Tumor resection	Unknown	None	Unknown	Alive
		Cardiomegaly						
Ono et al., 2007 [10]	20 years	Dyspnea	Multiple	Tumor resection	No	None	None	Alive
This case	1 day	Tachypnea	Multiple	None	Yes	*TSC2*	Depigmented nevus	Alive
		Tachycardia					Adenoma sebaceum	
		Cardiomegaly					Depigmented retina	
							Developmental delay	

*TSC2*: tuberous sclerosis 2.
